# Biochar-based fertilizer increases soil nutrients and enhances tea quality: a metabolomics-based analysis

**DOI:** 10.3389/fpls.2025.1552759

**Published:** 2025-05-30

**Authors:** Zhenyu Yang, Zetao Ren, Xiangzhen Zhu, Wenyan Yang, Zhengqian Ye, Luming Tian, Jiawei Ma

**Affiliations:** ^1^ College of Tea Science and Tea Culture, Zhejiang A & F University, Hangzhou, Zhejiang, China; ^2^ College of Resources and Environment, Zhejiang A & F University, Hangzhou, Zhejiang, China; ^3^ Soil, Fertilizer, Plant Protection and Energy Center of Qingyuan Agriculture and rural Bureau, Lishui, Zhejiang, China

**Keywords:** biochar-based fertilizer, soil nutrients, tea plant growth, tea quality, metabolomics

## Abstract

**Introduction:**

Biochar-based fertilizers (BF) have emerged as a promising strategy to improve soil physicochemical and biological properties, thereby enhancing tea yield and quality.

**Methods:**

A field experiment was conducted using two types of BF- ordinary BF (BF1, containing 15% biochar) and optimized BF (BF2, containing 30% biochar)- applied for either 1 or 2 years. The effects on the soil nutrients, tea plant growth, tea quality, and metabolomics profiles were assessed.

**Results:**

Results showed that BF application significantly increased soil pH and nutrient availability, as well as the bud length and hundred-bud weight (*p* < 0.05). Notably, BF2 applied for 2 years significantly increased the content of free amino acids, total flavonoids, soluble sugar, while reduced the phenol-ammonia ratio (*p* < 0.05), thereby improving tea quality. Further metabolomics analysis revealed that BF2 treatment significantly elevated the levels of amino acids, including theanine, threonine, proline, valine, and glutamic acid, while decreasing catechins including C, EC, and EGCG, thus leading to reduced bitterness and astringency and enhanced freshness. Besides, differential metabolites were mainly involved in amino acid and flavonoid biosynthesis pathway.

**Discussion:**

Taken together, prolonged BF2 application significantly improved soil fertility, promoted tea growth, and enriched flavor-related metabolites, offering valuable insights for optimizing fertilization strategies in tea plantations.

## 1 Introduction

Tea (*Camellia sinensis*) is a subtropical evergreen shrub with a long cultivation history in China (Zhang et al., 2023a). As a major leafy cash crops, tea represents a key agricultural industry and an important source of income for farmers in many developing countries. In 2023, China’s tea plantation area expanded to 3.43 million hectares, ranking first globally, with tea production of 3.54 million tons, accounting for over 50% of global production (National Bureau of Statistics, 2024; Peng et al., 2024). With rising market demand, farmers have intensified fertilizer application to maximize yield potential. However, excessive fertilizer application has led to several soil-related issues in tea gardens, including soil nutrient leaching and acidification. Such events not only lead to deficiencies in essential nutrients such as phosphorus (P), potassium (K), and magnesium (Mg), but also promote the accumulation of heavy metal elements in tea leaves (Dai et al., 2017a; Yan et al., 2018).

As a soil conditioner, biochar application can improve soil structure and water retention (Li et al., 2018), and enhances soil nutrient availability and organic matter content (Ullah et al., 2020). Previously, Yang et al. (2021) compared the effects of biochar and biochar-based fertilizer on soil fertility, tea yield and quality. The results indicated that biochar-based fertilizer significantly increased tea yield and quality, and led to higher soil pH, TN, AP, and AK content compared to pure biochar. Other studies have shown that, compared with the application of bamboo charcoal, the combined use of bamboo charcoal and organic fertilizer significantly improved soil TN content as well as the activities of sucrase, and beta-glucosidase (Zhang et al., 2023b). However, once biochar is applied to the soil, its effects are largely irreversible. This permanence poses potential risks during its application. Studies have shown that biochar may release heavy metals and organic pollutants, which can adversely affect plant growth and reduce crop yields. Additionally, although biochar possesses strong adsorption capacity, it lacks selectivity. As a result, it may also adsorb essential nutrients and microelements, potentially decreasing the availability of biologically active substances for soil microorganisms (Coughlan et al., 2002). Biochar-based fertilizer (BF) is a novel type of organic fertilizer that has been increasingly used in agricultural production in recent years (Zhang et al., 2019). It is composed of biochar and organic fertilizer, and has been shown to improve soil properties, such as the pH and nutrient content, thereby contributing to increased crop yield and quality (Zhang et al., 2020). Studies have shown that the combined application of biochar and organic fertilizer increased SOM, TN, TP, Ca, Mg content, and microbial biomass carbon and nitrogen, thus significantly increased maize yield (Wang et al., 2022). Hou et al. (2024) reported that this combination also enhanced, primarily by increasing the diversity of bacterial communities in the rhizosphere. Similarly, Chen et al. (2023) found that BF application improved soil fertility and enzymatic activity, enhanced photosynthetic capacity, and ultimately increased both yield and sugar content of sugar beets. In another study, Yin et al. (2022) investigated the influences of various BF application rates on plant behavior, and reported that 600 kg·hm^−2^ of BF significantly improved maize dry matter accumulation, ear length, plant height, and yield. These findings collectively suggest that appropriate BF application enhances rhizosphere soil conditions, promotes balanced biomass distribution, and increases crop production.

Plant metabolomics involves the qualitative and quantitative analysis of low-molecule metabolites in plants (Sun et al., 2022; Wan et al., 2023). As an integrative technique combining advanced analytical chemistry with chemoinformatic, it enables the identification of key differential metabolites and elucidate underlying metabolic pathways and regulatory mechanisms (Li et al., 2021). Metabolomics can be categorized into targeted and untargeted metabolomics, employing primarily mass spectrometry and nuclear magnetic resonance technologies (Wen et al., 2023). Untargeted metabolomics, characterized by high throughput, ultra-sensitivity, broad metabolite coverage and qualitative accuracy, has been extensively applied in tea quality assessment. This approach facilitates a comprehensive understanding of tea metabolite profiles and quality determinants (Dai et al., 2017b). Moreover, UHPLC-Q Exactive HF-X platform has been widely employed for such analyses due to its superior resolution, high quality and accuracy, particularly in the evaluation of non-volatile components in tea (Wen et al., 2023).

Numerous studies have demonstrated the beneficial effects of BF application in alleviating soil acidification and improving soil properties. However, most of these studies primarily focused on grain and vegetable crops, with limited attention given to their impacts on tea plantation. In particular, how the type of BF and the duration of its application influence soil health and tea quality remains poorly understood. Moreover, the specific impacts of BF on the accumulation of tea secondary metabolites have rarely been reported. To address these gaps, this study evaluates the effects of different types of BF and application durations on tea plant growth, leaf quality and secondary metabolite profiles. The findings aim to provide a theoretical basis for optimizing BF use in tea cultivation and for enhancing tea quality.

## 2 Materials and methods

### 2.1 Experimental design

The experimental site was located in Huizhou Village, Changxing City, Zhejiang Province, China (119°52’16.313”E, 31°07’06.254”N), situated in the center of the Yangtze River Delta. The region lies in a transitional area between the low hills of northern Zhejiang and the western plain Taihu Lake, with a west-to-east descending gradient. The area experiences a subtropical maritime monsoon climate, characterized by concentrated rainfall from March to September, with a mean annual precipitation of 1,309 mm. The average annual temperature is 15.6°C, with 1,810.3 hours of annual sunshine annually, and an elevation of approximately 55 meters above sea level. In 2021, tea plantations with relatively uniform growth potential were selected for this study. The soil type was lateritic red soil, with the following baseline chemical properties: soil pH 4.05, SOM 3.2%, AN 120.00 mg/kg, AP 12.12 mg/kg, and AK 162.50 mg/kg. The agricultural management of the tea garden includes fertilization in November, spring tea picking in April of the following year, and pruning in May.

The BF used in this study was provided by Zhejiang Yangtze River Delta Poly Agricultural Technology Development Co., Ltd. The BF was produced by wood chip-derived biochar as the primary raw material, with biochar and organic fertilizer mixed in a specific proportion. Among them, BF1 is an ordinary biochar-based fertilizer with 15% biochar content, while BF2 is an optimized biochar-based fertilizer with 30% biochar content. The organic fertilizer used was straw organic fertilizer, and the basic physical and chemical properties of BF are shown in **Supplementary Table S1**. The experimental treatments were as follows: CK: conventional fertilization; T1: conventional fertilization + BF1 application for 1 year; T2: conventional fertilization + BF1 application for 2 years; T3: conventional fertilization + BF2 application for 1 year; T4: conventional fertilization + BF2 application for 2 years. Consistent with conventional practice in tea plantation, the BF was applied in ditches (~20 cm in depth and ~25 cm in width) between plant rows. The application rate for each treatment was 3,000 kg/ha. Each treatment included three replicates arranged in a randomized complete block design, with each plot covering 60 m^2^. The field experiments were conducted continuously for 1 and 2 years. To ensure the normal growth of tea trees, all field management, including pest control, were kept consistent across treatments.

### 2.2 Sample collection and processing

All tea samples were collected on April 1, 2023, following the manual picking standard of one bud and two leaves, and the tea bud length and hundred-bud weight were immediately determined (Huang et al., 2023). The remaining samples were freeze-dried according to Fang et al. (2023) for the determination of tea quality indicators, and another portion of the samples was immediately frozen in liquid nitrogen for subsequent metabolomics analysis.

Water extract content was measured according to the National standard methods of GB/T 8305–2013, established by China National Institute of Standardization (CNIS). The tea polyphenols content was determined by Folin-Ciocalteu reagent at 765 nm with a UV1800PC ultraviolet–visible (UV–vis) spectrophotometer (Shanghai Jinghua Technologies, Shanghai, China), based on the CNIS GB/T 8313–2013. Free amino acids content was measured at 570 nm using ninhydrin assay, in accordance with the CNIS GB/T 8314–2013. Caffeine content was determined based on the CNIS GB/T 8312–2013. The phenol-ammonia ratio was expressed as the ratio of tea polyphenols content to free amino acid content. Total flavonoids in tea were determined by aluminum trichloride-sodium nitrite colorimetry method, with rutin as the standard (Liu et al., 2018). The content of soluble protein in tea was quantified by Coomassie Brilliant Blue colorimetric method, with bovine serum albumin as the standard (Zhao et al., 2020), and the content of soluble sugar was determined by anthrone reagent at 620 nm, following extraction with 80% (*v/v*) ethanol solution at 50°C for 20 min (Ma et al., 2021).

Soil samples from the 0–20 cm soil layers of tea garden were collected from each experimental plot using the five-point sampling method. Soil samples were naturally air-dried, cleaned of stones and plant roots, ground, and passed through a 100-mesh (0.15 mm) sieve to ensure homogeneity for subsequent chemical analysis. Following the methods described by Carter and Gregorich (2007), soil pH (soil: water, 1:2.5 *w/v*) was measured using a standard pH meter. Soil organic matter content was determined by H_2_SO_4_ - K_2_Cr_2_O_7_ titration (Kalembasa and Jenkinson, 2006). Available nitrogen was analyzed by alkaline hydrolysis diffusion method using NaOH (Bao, 2000). Available phosphorus was extracted using NaHCO_3_ solution and then quantified by the molybdenum-antimony colorimetric method, while available potassium was extracted with NH_4_OAC solution and analyzed by flame photometer (Bao, 2000). For each treatment, leaf and soil samples were randomly collected with three biological replicates per group.

### 2.3 Tea metabolomic determination

As previously described by Fang et al. (2023), 50 ± 5mg of tea samples was accurately weighed into a 2 mL centrifuge tube. A 6 mm diameter grinding bead and 400 µL of extraction solution (methanol: water = 4:1 (v: v)) containing four internal standards (L-2-chlorophenylalanine (0.02 mg/mL), etc.) were added. The samples were grounded using a frozen tissue grinder for 6 min (-10°C, 50 Hz), followed by low-temperature ultrasonic extraction for 30 min (5°C, 40 KHz). After standing at -20°C for 30 min, the samples were centrifuged for 15 min (13000 g, 4°C), and the supernatant was then transferred into an injection vial with an inner cannula for analysis. Additionally, 20 µL of supernatant from each sample was mixed as a quality control sample.

The LC-MS analysis was performed using a Thermo Fisher’s UHPLC-Q Exactive HF-X system, combining ultra-high performance liquid chromatography tandem with Fourier transform mass spectrometry. Chromatographic conditions were as follows: the column employed was ACQUITY UPLC HSS T3 (100 mm × 2.1 mm i.d., 1.8 µm; Waters, Milford, USA); mobile phase A was 95% water + 5% acetonitrile (0.1% formic acid), mobile phase B was 47.5% acetonitrile + 47.5% isopropanol + 5% water (0.1% formic acid), the injection volume was 3 μL, and the column temperature was set at 40°C. The samples were ionized by electrospray ionization, and the mass spectrometry signals were collected in positive and negative ion scanning modes. The specific parameters were as follows: scan range of 70–1050 m/z, sheath gas flow rate of 50arb, auxiliary gas flow rate of 13arb, heater temperature of 425°C, capillary temperature of 325°C, spray voltage of 3500V (positive mode), and -3500V (negative mode), S-Lens voltage of 50, collision energy settings of 204060eV, a full MS resolution of 6000, and an MS/MS resolution of 7500 (Fang et al., 2023).

### 2.4 Statistical analysis

SPSS 26.0 software was used for statistical analysis, and Origin 2021 software was used to generate figures. All results were presented as mean ± standard deviation. One-way analysis of variance (ANOVA) and Duncan multiple comparison method were used to assess differences among treatments, with statistically significant differences indicated by different letters (*p* < 0.05).

The R package “ropls” (Version 1.6.2) was used to perform principal component analysis (PCA) and partial least squares-discriminant analysis (PLS-DA). Differential metabolite-associated pathways were annotated based on the KEGG database (http://www.genome.jp/kegg/).

## 3 Results

### 3.1 Effects of BF1 and BF2 on soil pH value and nutrient content

**Figure 1:**
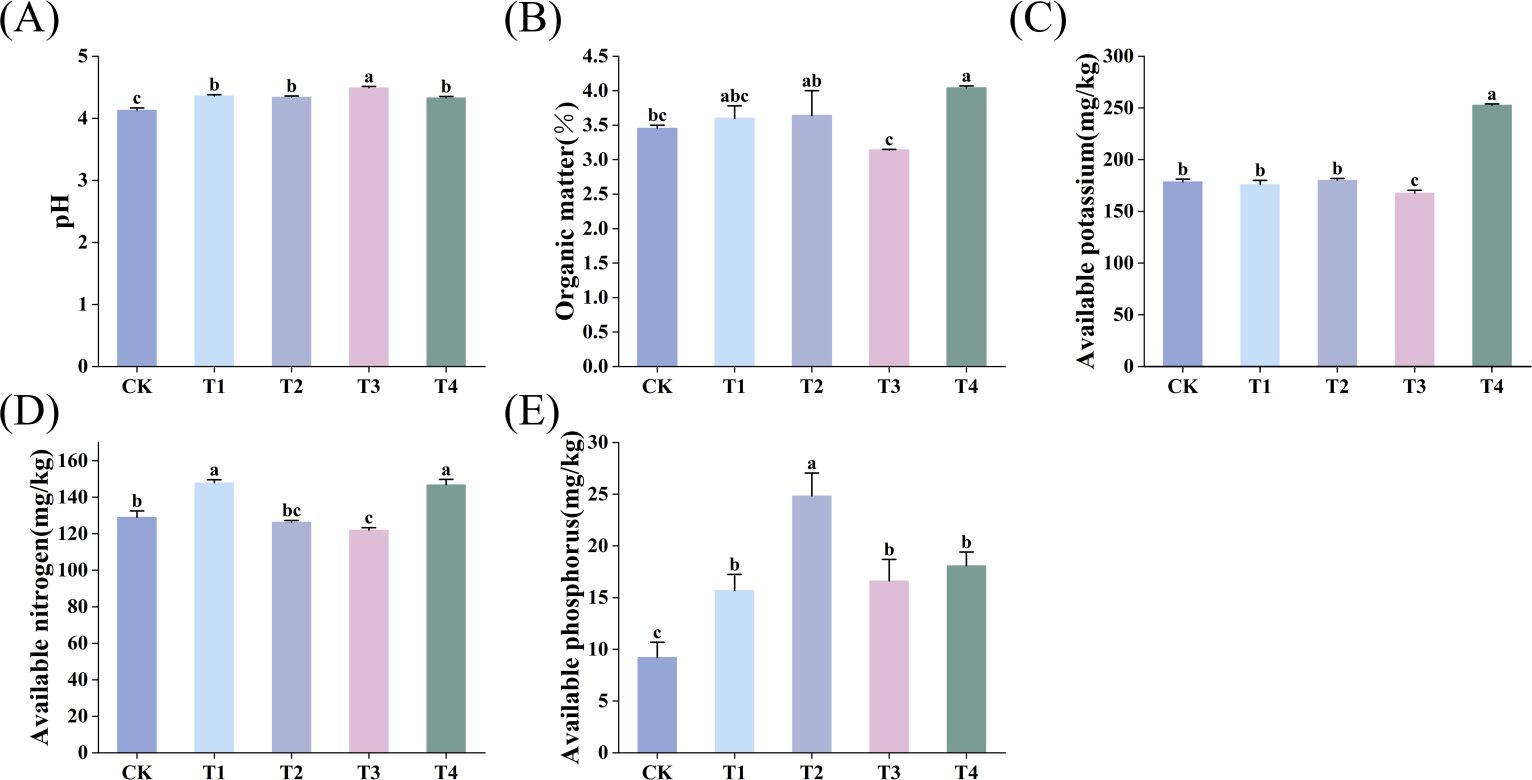
Effects of different BF treatments on soil nutrients (CK, conventional fertilization; T1, conventional fertilization + BF1 application for 1 year; T2, conventional fertilization + BF1 application for 2 years; T3, conventional fertilization + BF2 application for 1 year; T4, conventional fertilization + BF2 application for 2 years). **(A)** pH, **(B)** Organic matter, **(C)** Available potassium, **(D)** Available nitrogen, **(E)** Available phosphorus. Different lowercase letters indicate significant differences between different treatments (p < 0.05).

Soil organic matter (SOM), nitrogen (N), phosphorus (P), and potassium (K), and pH are critical indicators of soil health and fertility (Zhu et al., 2021). As shown in **Figure 1**, BF treatments significantly influenced soil nutrient contents. Compared with conventional fertilization (CK), both types of BF and different application durations significantly increased soil pH, with significant differences observed among treatments (*p* < 0.05). The most pronounced increase in pH (8.71%) was observed in the optimized biochar-based fertilizer (BF2) treatment after 1 year (T3) (**Figure 1A**). Compared to CK, T1 and T2 treatments did not significantly affect SOM and available potassium (AK) content (*p* > 0.05). However, the contents of SOM and AK significantly increased with longer BF2 application periods (*p* < 0.05), with T4 (BF2 applied for 2 years) showing the greatest increases in SOM (17.10%) and AK (41.52%) (*p* < 0.05) (**Figures 1B, C**). For soil available nitrogen (AN) (**Figure 1D**), the application of ordinary biochar-based fertilizer (BF1) for 1 year and BF2 for 2 years resulted in the highest improvements (14.53% and 13.71%, respectively; *p* < 0.05). For soil available phosphorus (AP) (**Figure 1E**), BF1 applied for 2 years led to the greatest enhancement (169.27%, *p* < 0.05), both BF1 and BF2 showed a time-dependent improvement. Overall, these findings indicate that BF application significantly increase SOM, AN, AP, AK and pH value, thus improving soil nutrient quality in tea plantations.

### 3.2 Effect of BF application on tea growth, quality and metabolomics

#### 3.2.1 Effects of BF application on tea bud length and hundred-bud weight

**Figure 2:**
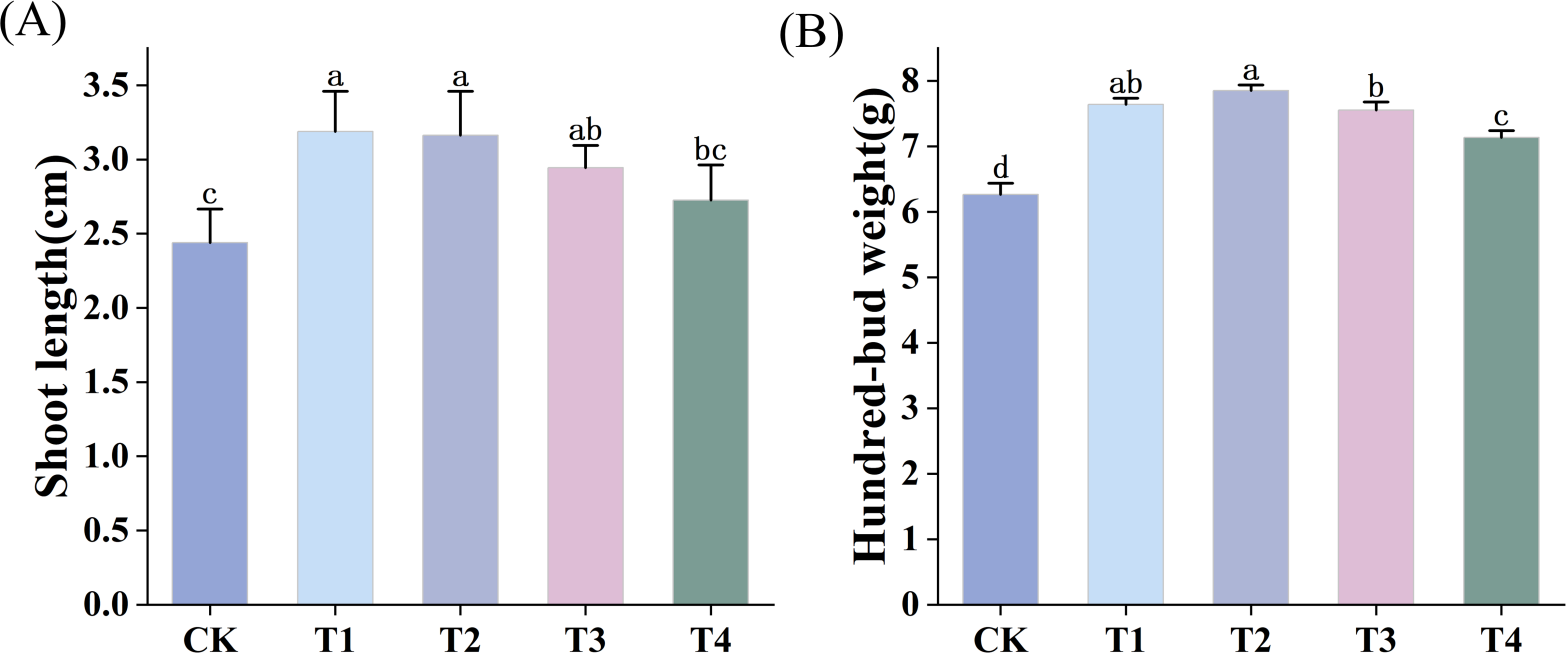
Effects of different BF treatments on shoot length and hundred-bud weight of tea leaves (CK, conventional fertilization; T1, conventional fertilization + BF1 application for 1 year; T2, conventional fertilization + BF1 application for 2 years; T3, conventional fertilization + BF2 application for 1 year; T4, conventional fertilization + BF2 application for 2 years). **(A)** Shoot length, **(B)** Hundred-bud weight. Different lowercase letters indicate significant differences between different treatments (p < 0.05).

Tea bud length and hundred-bud weight are key indicators of tea yield. As shown in **Figure 2**, different BF and application durations influenced these parameters to varying degrees compared with CK. The application of BF1 for 1 year (T1) and 2 years (T2) resulted in the greatest increase in tea bud length (30.74% and 29.51%, respectively), while the difference between T1 and T2 was not statistically significant (*p* > 0.05). The effects of BF2 application for 1 year (T3) and 2 years (T4) on tea bud length were lower than that of T1 and T2 treatments, with increases of 20.49% and 11.89%, respectively (**Figure 2A**). Similarly, T1 and T2 treatments also achieved the largest increase in tea hundred-bud weight (22.04% and 25.40%, respectively). Although T2 showed a slightly greater increase than T1, the difference was not statistically significant (*p* > 0.05). The increases under T3 and T4 treatments were lower compared to T1 and T2. Taken together, BF1 application resulted in a greater effect on improving tea bud length and hundred-bud weight than BF2, though differences between different application years were not statistically significant (*p* > 0.05). These findings suggest that BF application can effectively improve tea plant growth and contribute to higher yields potential.

#### 3.2.2 Effects of BF application on tea quality

**Figure 3:**
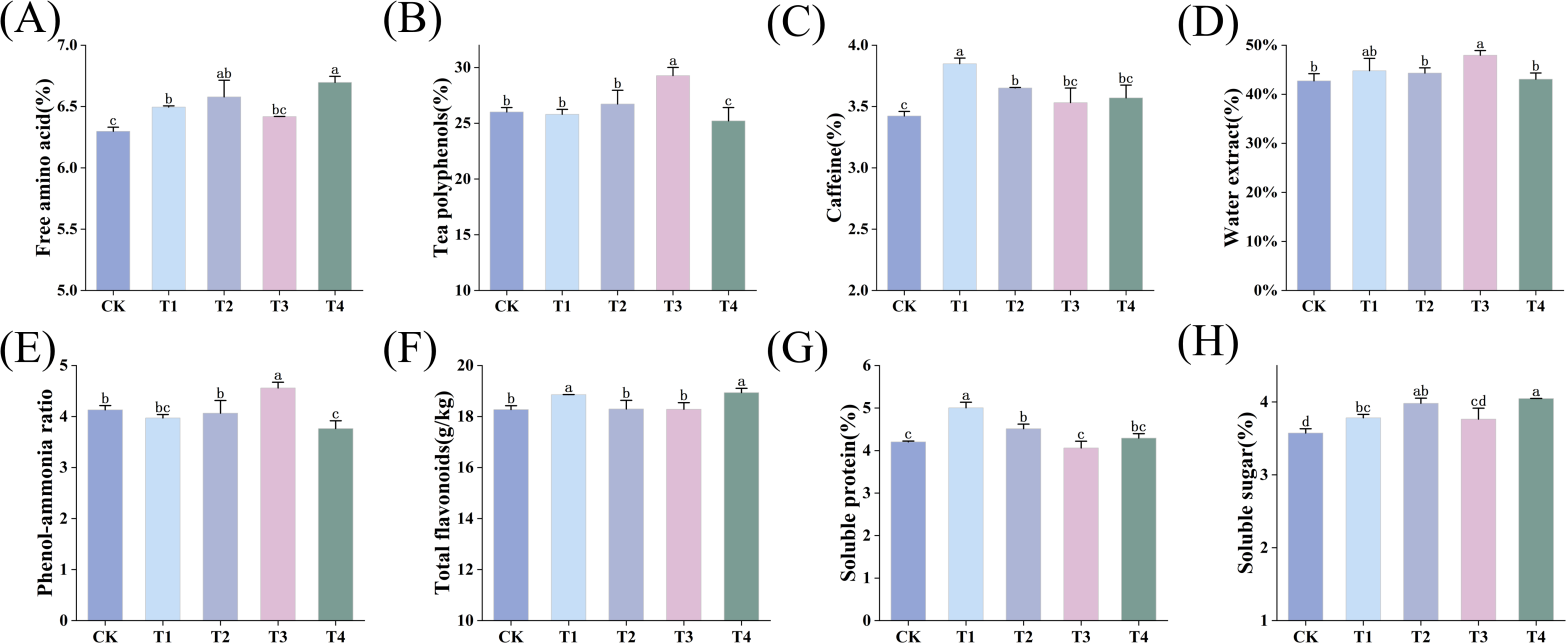
Effects of different BF treatments on tea quality (CK, conventional fertilization; T1, conventional fertilization + BF1 application for 1 year; T2, conventional fertilization + BF1 application for 2 years; T3, conventional fertilization + BF2 application for 1 year; T4, conventional fertilization + BF2 application for 2 years). **(A)** Free amino acid, **(B)** Tea polyphenols, **(C)** Caffeine, **(D)** Water extract, **(E)** Phenol-ammonia ratio, **(F)** Total flavonoids, **(G)** Soluble protein, **(H)** Soluble sugar. Different lowercase letters indicate significant differences between different treatments (p < 0.05).

Tea polyphenols, free amino acids, caffeine, the phenol-ammonia ratio, total flavonoids and soluble sugar are important indicators for evaluating tea quality. Compared with CK, the application of different BF improved the free amino acid content of tea leaves in different duration of application. Among all treatments, BF2 application for 2 years resulted in the greatest increase in the free amino acid content (6.4%), followed by BF1 application of for 2 years, indicating that the improvement enhanced with prolonged application (**Figure 3A**). In terms of tea polyphenols content, T1 and T2 treatments showed no significant differences compared with the CK treatment (*p* > 0.05). However, the application of BF2 had a significant impact depending on the duration (*p* < 0.05). Specifically, BF2 application for 1 year significantly increased the content of tea polyphenols (*p* < 0.05), whereas application for 2 years significantly decreased this value (*p* < 0.05) (**Figure 3B**). Regarding caffeine content, BF2 application had no significant effect compared to CK across different years (*p* > 0.05). In contrast, BF1 application significantly increased the caffeine content of tea leaves (*p* < 0.05). with the greatest enhancement observed after 1 year of application (12.57%), followed by 2 years (**Figure 3C**).

Only the T3 treatment significantly increased the content of water extract in tea leaves by 12.19% compared to CK (*p* < 0.05) (**Figure 3D**). BF2 application for 2 years (T4) significantly reduced the tea phenol-ammonia ratio (*p* < 0.05) (**Figure 3E**). No significant differences were observed between the T1, T2, and CK treatment (*p* > 0.05); however, T3 treatment showed an upward trend, suggesting that T4 treatment was more favorable for enhancing tea freshness. The application of BF1 across different years significantly increased the soluble protein content (*p* < 0.05) (**Figure 3G**), whereas BF2 showed no significant effect (*p* > 0.05). Compared to CK treatment, BF2 application for 2 years (T4) had the most pronounced effect on increasing total flavonoids and soluble sugar content (**Figures 3F, H**). Overall, BF application exerted varied effects on tea quality, with BF2 demonstrating superior improvements after 2 years of application.

#### 3.2.3 Metabolomic analysis

##### 3.2.3.1 Principal component analysis and partial least squares discriminant analysis

**Figure 4:**
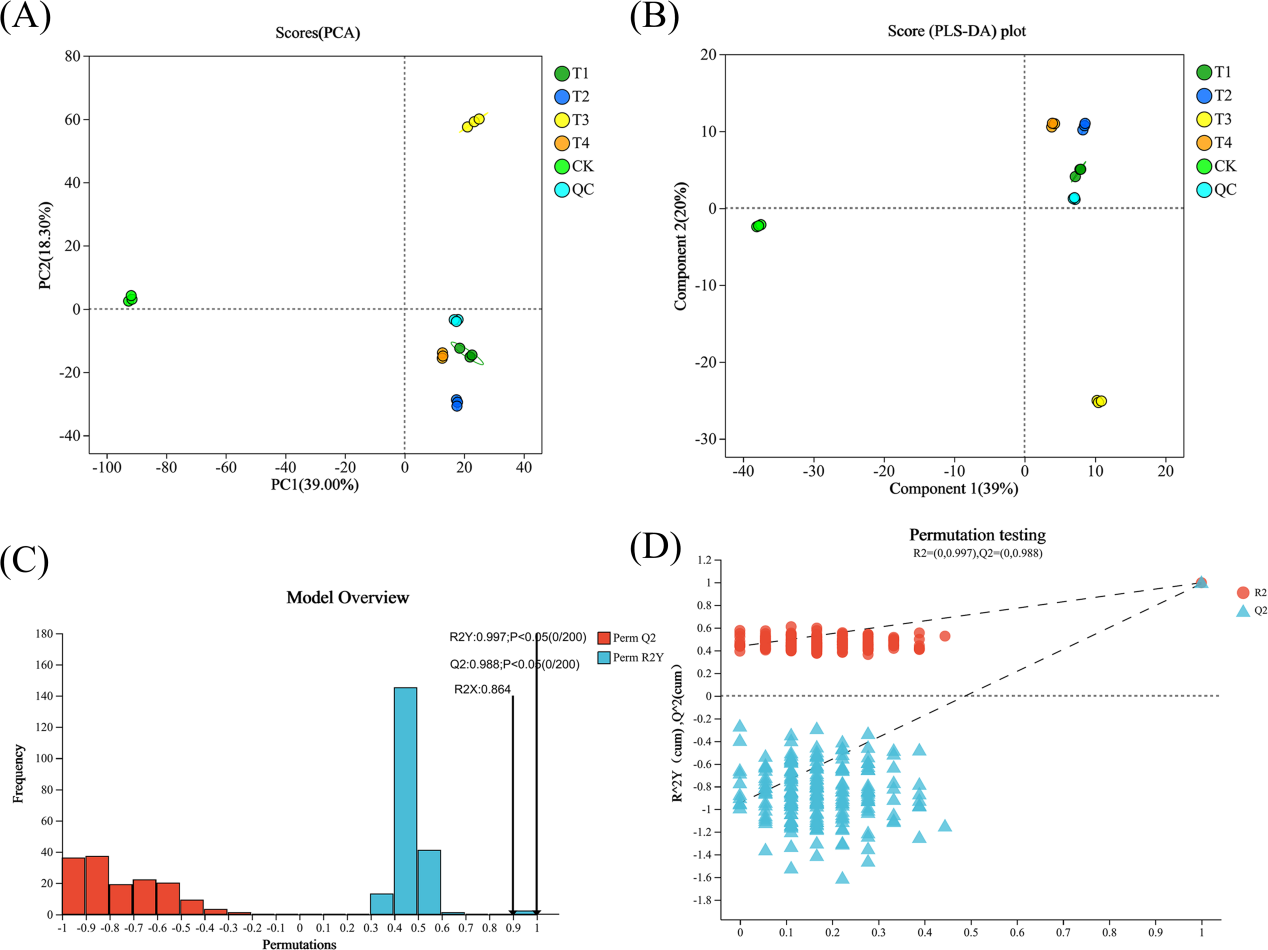
PCA **(A)**, PLS-DA **(B)**, model test results **(C, D)** of tea metabolites under different fertilization conditions (CK, conventional fertilization; T1, conventional fertilization + BF1 application for 1 year; T2, conventional fertilization + BF1 application for 2 years; T3, conventional fertilization + BF2 application for 1 year; T4, conventional fertilization + BF2 application for 2 years).

To elucidate the metabolic responses of tea leaves to different BF treatments and application durations, untargeted metabolomics using LC-MS was used to identify metabolites associated with BF application. Principal Component Analysis (PCA), a multivariate statistical analysis method for unsupervised pattern recognition, was used to analyze the metabolite profiles. Differences in metabolites across samples are observed in the scatter plot of the sample distribution. The distance between samples in the plot corresponds to the dissimilarity in their metabolite profiles, with larger distances indicating greater metabolic differences. As shown in **Figure 4A**, significant separation between treatments were observed, particularly along PC1, which accounted for 39% of the variation, indicating considerable metabolic variation between the groups.

Partial Least Squares Discriminant Analysis (PLS-DA) is a supervised discriminant analysis method used for classification and prediction. The R2X and R2Y values represent the explained variance of the X and Y matrices, respectively, while Q2 indicates the predictive ability of the model. Higher cumulative values suggest a more stable and reliable model. The score plot of tea metabolites, obtained through PLS-DA analysis, is shown in **Figure 4B**. The results showed that R2X, R2Y and Q2 values were all close to 1, and Q2 > 0.9, *p* < 0.05, indicating a satisfactory model fit. As shown in **Figures 4C, D**, CK and T3 treatments were grouped into one category, while T1, T2, and T4 treatments were classified into another. Following 200 permutation tests, the R2 and Q2 values from random permutations were consistently lower than those from the original model. Additionally, the steep slope of the regression line further validated that the PLS-DA model was not overfitted, confirming its reliability.

##### 3.2.3.2 Differential metabolite screening

**Figure 5:**
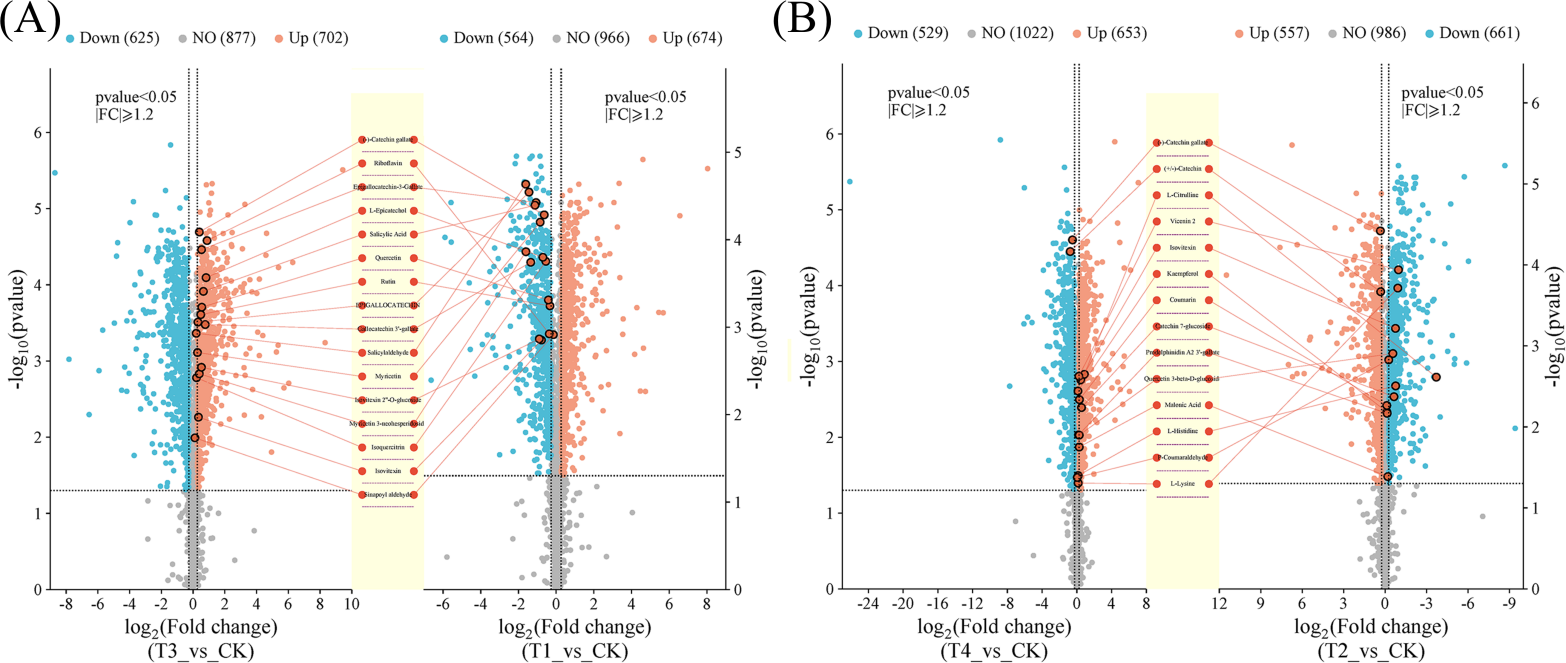
Volcanic map of differential metabolites (CK, conventional fertilization; T1, conventional fertilization + BF1 application for 1 year; T2, conventional fertilization + BF1 application for 2 years; T3, conventional fertilization + BF2 application for 1 year; T4, conventional fertilization + BF2 application for 2 years). **(A)** T3 vs CK and T1 vs CK, **(B)** T4 vs CK and T2 vs CK.

**Figure 6:**
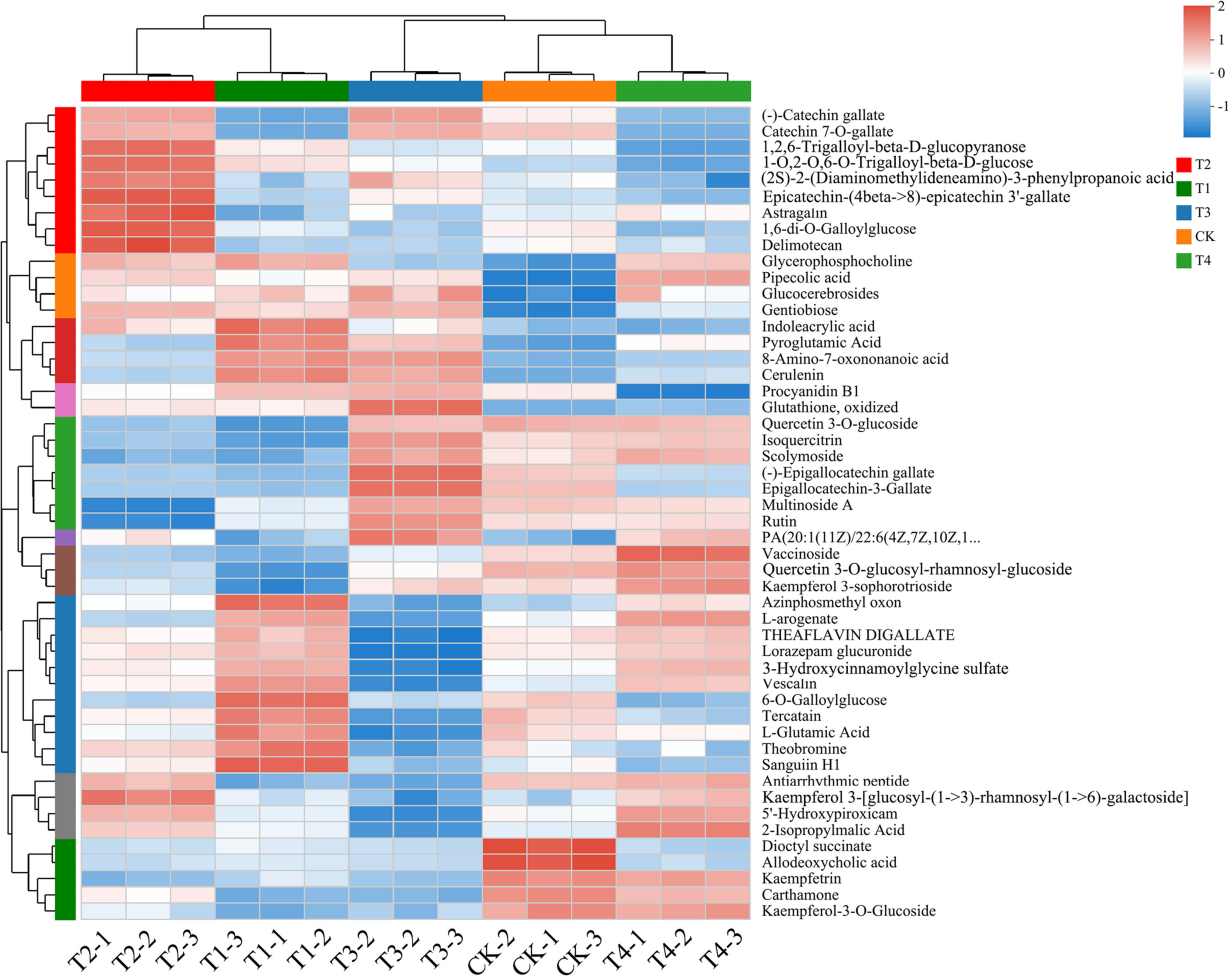
Heat map of aggregate metabolite cluster analysis (CK, conventional fertilization; T1, conventional fertilization + BF1 application for 1 year; T2, conventional fertilization + BF1 application for 2 years; T3, conventional fertilization + BF2 application for 1 year; T4, conventional fertilization + BF2 application for 2 years).

Differential metabolites were screened based on the variable importance in projection (VIP) from the PLS-DA model, combined with the fold change (FC) and *p* < 0.05. Using the screening criteria of VIP > 1, *p* < 0.05 and | log_2_FC | ≥ 1, a total of 1238 differential metabolites (674 up-regulated and 564 down-regulated) were found between T1 and CK (**Figure 5A**), while 1218 differential metabolites (557 up-regulated and 661 down-regulated) were identified between T2 and CK (**Figure 5B**). Additionally, 1327 (702 up-regulated and 625 down-regulated) and 1182 (653 up-regulated and 529 down-regulated) differential metabolites were found between the T3 *vs*. CK and T4 *vs*. CK, respectively (**Figures 5A, B**). Notably, more up-regulated metabolites were observed than down-regulated ones across different fertilization treatments, with T3 exhibiting the highest number of up-regulated metabolites. To gain a more intuitive understanding of the changes in differential metabolites between across BF types and application years, cluster analysis was performed on the differential metabolites shared among the five control groups (**Figure 6**).

##### 3.2.3.3 Metabolite content

**Figure 7:**
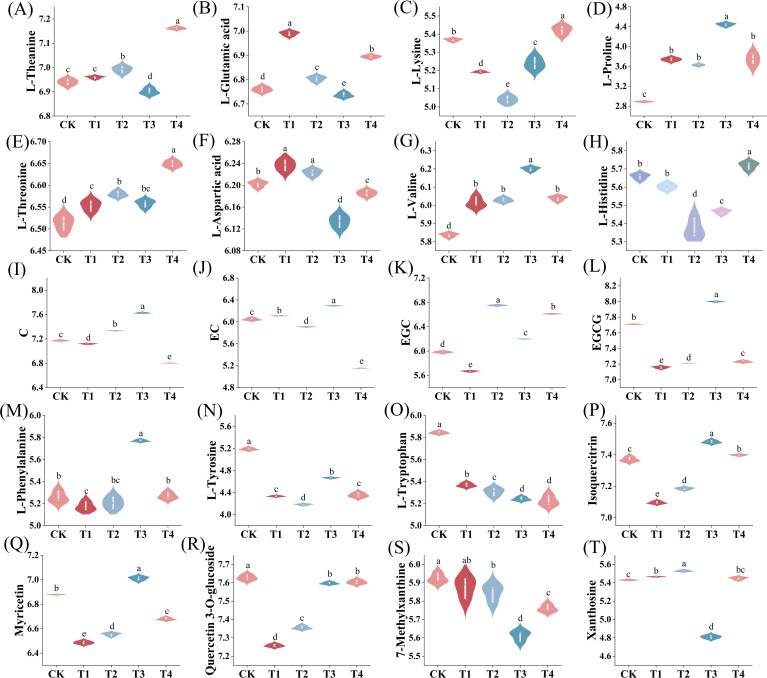
Relative content of metabolites under different treatments (CK, conventional fertilization; T1, conventional fertilization + BF1 application for 1 year; T2, conventional fertilization + BF1 application for 2 years; T3, conventional fertilization + BF2 application for 1 year; T4, conventional fertilization + BF2 application for 2 years). **(A)** L-Theanine, **(B)** L-Glutamic acid, **(C)** L-Lysine, **(D)** L-Proline, **(E)** L-Threonine, **(F)** L-Aspartic acid, **(G)** L-Valine, **(H)** L-Histidine, **(I)** C, **(J)** EC, **(K)** EGC, **(L)** EGCG, **(M)** L-Phenylalanine, **(N)** L-Tyrosine, **(O)** L-Tryptophan, **(P)** Isoquercitrin, **(Q)** Myricetin, **(R)** Quercetin-3-O-glucoside, **(S)** 7-Methylxanthine, **(T)** Xanthosine. Different lowercase letters indicate significant differences between different treatments (p < 0.05).

As shown in **Figure 7**, the application of different BF types and durations significantly affected the levels of various amino acids and catechins in tea compared to the CK treatment. BF application generally increased the content of most tea amino acids, including theanine, threonine, valine, and proline, although the magnitude of increase varied. Specifically, BF application for 2 years (T4) resulted in the greatest improvement in the content of theanine, lysine, threonine, and histidine. In addition, significant differences in catechin content were observed across treatments. The contents of C, EC and EGCG were highest under T3 treatment, while the lowest levels were observed under T4. For EGC, the content was significantly higher in both T2 and T4 treatments compared to other treatments.

##### 3.2.3.4 Differential metabolite KEGG pathway enrichment

**Figure 8:**
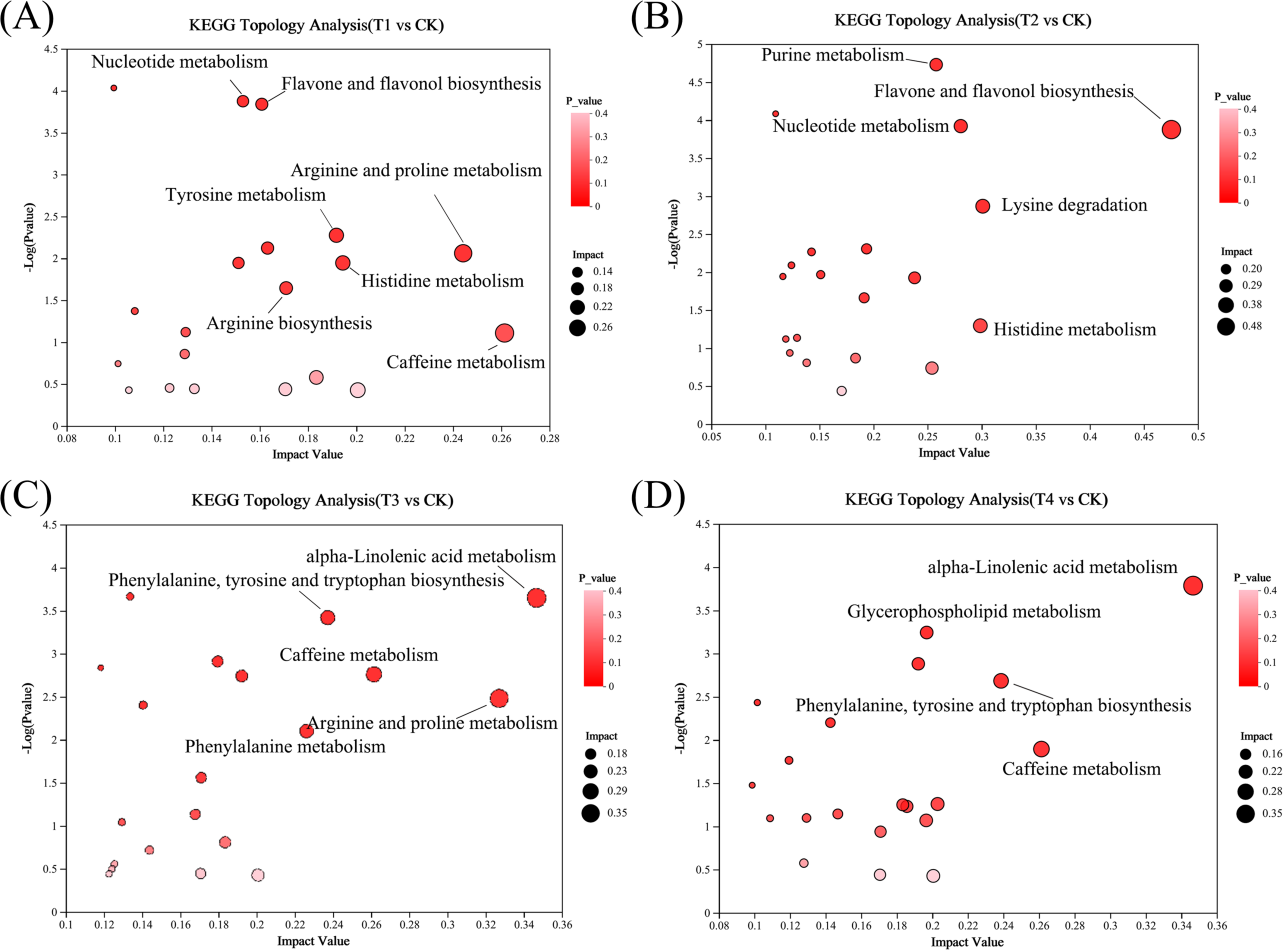
KEGG enrichment analysis of differential metabolites in different treatment groups (CK, conventional fertilization; T1, conventional fertilization + BF1 application for 1 year; T2, conventional fertilization + BF1 application for 2 years; T3, conventional fertilization + BF2 application for 1 year; T4, conventional fertilization + BF2 application for 2 years). **(A)** T1 vs CK, **(B)** T2 vs CK, **(C)** T3 vs CK, **(D)** T4 vs CK.

KEGG pathway analysis was conducted to identify the key metabolic pathways in tea under different BF types and application durations. To directly compare metabolic pathways differences between treatment and control group, the most enriched pathways in each contrast group were selected through enrichment and topological analysis. In the T1 *vs*. CK comparison, significant enrichment was observed in pathways related to arginine and proline metabolism, flavone and flavonol biosynthesis, and histidine metabolism (**Figure 8A**). In the T2 *vs*. CK comparison, pathways involved in flavone and flavonol biosynthesis, lysine degradation, and nucleotide metabolism were significantly enriched (**Figure 8B**). For T3 *vs*. CK, significant enrichment was found in pathways such as α-linolenic acid metabolism, arginine and proline metabolism, phenylalanine, tyrosine and tryptophan biosynthesis, and caffeine metabolism (**Figure 8C**). For the T4 *vs*. CK comparison, the most significantly enriched pathways included α-linolenic acid metabolism, caffeine metabolism, phenylalanine, tyrosine and tryptophan biosynthesis, and glycerophospholipid metabolism (**Figure 8D**).

In the biosynthesis pathway of phenylalanine, tyrosine and tryptophan, BF1 and BF2 applied for 2 years had no significant effect on phenylalanine content (**Figure 7M**) (*p* > 0.05), while BF2 applied for 1 year significantly increased phenylalanine content compared to CK (*p* < 0.05). Additionally, BF1 application significantly reduced the content of tyrosine (**Figure 7N**) and tryptophan (**Figure 7O**) in tea (*p* < 0.05) compared to CK. Among these, BF1 applied for 2 years had the most significant effect on reducing tyrosine, while BF2 exhibited the most significant reduction in tryptophan content. In the biosynthesis pathway of flavonoids and flavonols, the content of isoquercitrin, myricetin, and quercetin 3-*O*-glucoside was lowest under BF1 applied for 1 year (**Figures 7P–R**). Conversely, BF2 applied for 1 year significantly increased the content of isoquercetin and myricetin compared to CK (*p* < 0.05).

## 4 Discussion

### 4.1 Effect on soil properties

Biochar-based fertilizers (BF) are known for their high nutrient retention capacity and soil amelioration effects, and have been widely used in agricultural production to improve soil fertility, as well as improve tea yield and quality. Soil pH is a fundamental physical and chemical property that is essential for evaluating soil health (Chen et al., 2022). The results showed that BF application increased soil pH (Wang et al., 2017), which is consistent with our findings. We hypothesize that BF is alkaline in nature, with optimized biochar-based fertilizer (BF2) having a higher pH than ordinary biochar-based fertilizer (BF1), leading to a more pronounced improvement with BF2. However, as cultivation duration increased, root exudate also increased, resulting in a decrease in soil pH. The optimal pH range for tea growth is 4.5-5.5, and an increase in soil pH in tea garden helps prevent nutrient loss and mitigates heavy metal toxicity (Yang et al., 2024). SOM is a core component of soil fertility, which can improve soil structure and provide essential nutrients for tea plant growth (Celestina et al., 2019). BF has been shown to increase SOM content (Yang et al., 2015), likely because biochar input slows the mineralization of organic matter and accelerates soil humification process over time (Lebrun et al., 2021). Biochar also adsorbs organic matter and contribute to soil humus formation (Kimetu and Lehmann, 2010), which in turn increases soil AN content. Additionally, BF contains certain nutrients such as P and K, which can directly supply nutrients to the soil. BF1 applied for 2 years significantly increased soil AP content, likely due to its higher P content and the gradual release of nutrients over time. This finding is in line with previous studies (Oladele et al., 2019; Zhou et al., 2019). The soil nutrient content and availability reflect the soil fertility, especially the availability of N, P, and K, which in turn influence the nutrient uptake capacity of plants (Mhlanga et al., 2022), as a higher content of available nutrients is beneficial for plant growth and yield (Oladele et al., 2019).

### 4.2 Tea plant growth and yield

Biochar combined with organic fertilizer has been shown to significantly improve the yields of red pitaya (Chen et al., 2023). Similarly, Yang et al. (2021) observed significant improvements in fresh tea product yield, hundred-bud weight, and tea sprouting density following the application of BF. The increase in tea biomass resulting from BF application is likely attributed to improved soil quality and enhanced plant nutrient availability. These improvements lead to higher photosynthetic rate and better growth parameters, ultimately providing more nutrients for the growth and development of tea plants (Azeez et al., 2010; Sun et al., 2024). Among the treatments, T1 and T2 were particularly effective in enhancing tea bud length and hundred-bud weight. This may be due to the relatively higher proportion of organic fertilizer in BF1 and the lower proportion of biochar. Studies shown that BF with a higher proportion of organic fertilizer tends to have a more favorable impact on crop yield (Chen et al., 2023).

### 4.3 Tea quality

Key determinants of tea quality include amino acids, tea polyphenols and the phenol-ammonia ratio. Amino acids contribute a refreshing flavor, while tea polyphenols impart bitterness, and the phenol-ammonia ratio serves as a critical factor for evaluating the taste of green tea, with a lower ratio reflecting higher quality (Zhang et al., 2021). In this study, tea leaves treated with BF2 for 2 years exhibited the highest amino acid content, highlighting the advantages of BF with a higher biochar proportion and prolonged duration. This improvement is likely due to the large specific surface area, rich nutrients, and high cation exchange capacity of biochar, which enhance the absorption and gradual release of essential elements (N, P, K) over time (Sun et al., 2024), thereby improving soil quality and supporting tea plant growth and quality formation (Chen et al., 2022). Meanwhile, the phenol-ammonia ratio of the BF2 treatment significantly decreased after 2 years of application, reaching the lowest level, which may be related to the higher biochar proportion in the BF2 (Martinetti and Paganini, 2006). Generally, the sweet taste of tea is attributed to TAAs and soluble sugar, and soil nitrogen availability is a key factor influencing soluble sugar content (Ruan et al., 2019a). The BF2 applied for 2 years enhanced soil AN content, thereby increasing soluble sugar content. Moreover, caffeine and water extract content, acting as the critical contributors to the bitterness and thickness of tea soup, were also improved (Mohanpuria et al., 2010). As secondary metabolites derived from photosynthesis, the biosynthesis of amino acids, caffeine, and water extract was enhanced under BF-induced improvement of tea photosynthetic performance (Liu et al., 2023). Additionally, BF application influenced soil microbial communities by recruiting beneficial bacteria, which positively correlated with tea yield and quality (Yan et al., 2020).

### 4.4 Metabolomic insights

**Figure 9:**
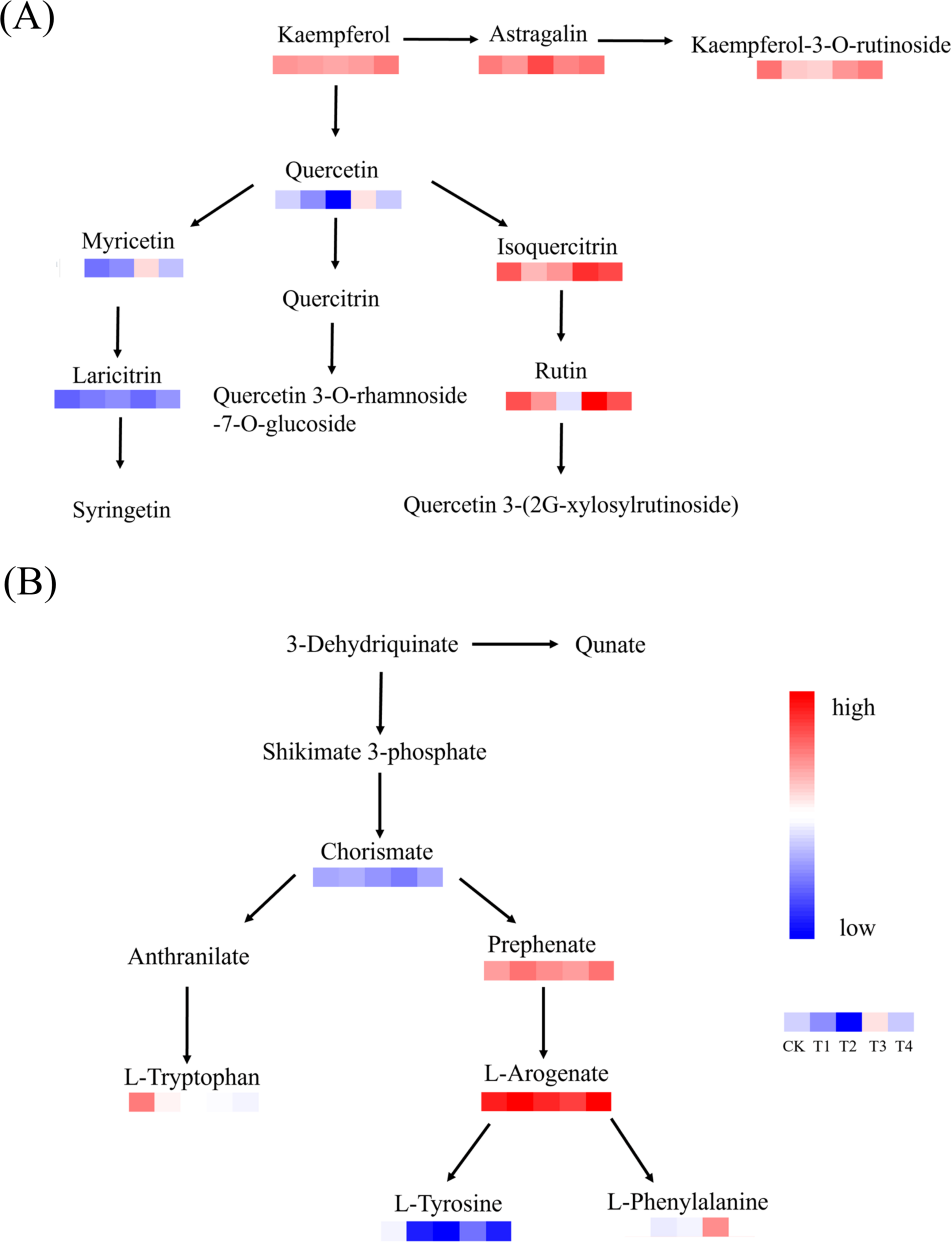
Map of major metabolic pathways under different biochar-based fertilizer. **(A)** Flavone and flavonol biosynthesis. **(B)** Phenylalanine, tyrosine and tryptophan biosynthesis.

This study identified 2204 metabolites in tea leaves, with carboxylic acids, their derivatives and flavonoid compounds accounting for the largest proportion. Catechins, key bioactive compounds in tea, offer a range of health benefits (Musial et al., 2020). As the main components of tea polyphenols, catechins represent 14-20% of the tea leaf dry matter. Different catechins show distinct taste profiles; for instance, EC contributes both bitterness and astringency, with bitterness being the more dominant sensation (Kallithraka et al., 1997). Similarly, EGCG is a major contributor to both the astringency and bitterness of tea soup (Yin et al., 2014). In this study, experimental treatments had differential effects on catechins content. Notably, BF2 applied for 1 year significantly increased the content of C, EC and EGCG in tea leaves, aligning with previous findings (Yin et al., 2024). This enhancement may be attributed to increased soil NO_3_^-^ availability, as fertilizers rich in NO_3_^-^ have been shown to promote greater catechin accumulation compared to those rich in NH_4_^+^ (Ruan et al., 2019b). KEGG enrichment analysis found that differential metabolites were mainly enriched in pathways related to amino acid biosynthesis, flavonoid and flavonol biosynthesis, and caffeine metabolism. Studies have shown that amino acids were particularly important for defining the taste quality of tea infusion (Chen et al., 2009; Zhu et al., 2016). Theanine, the most abundant amino acid in tea, imparts umami, bitterness, and sweetness, with flavor intensity positively correlated with its content (Horanni and Engelhardt, 2013). Additionally, threonine, valine, and proline contribute to sweetness for the formation of tea taste, while histidine enhances bitterness (Thippeswamy et al., 2006). BF application significantly increased the levels of several amino acids, thereby improving the tea freshness and quality. In the flavone and flavonol biosynthesis pathway, BF2 application significantly increased the levels of isoquercitrin and myricetin compared to other treatments, likely due to enhanced soil nutrient availability, thereby increasing secondary metabolites accumulation (Liu et al., 2023). In summary, differential metabolites were mainly enriched in flavonoid and flavonol biosynthesis (**Figure 9A**) and amino acid biosynthesis (**Figure 9B**). These findings suggest that BF application effectively increased the accumulation of secondary metabolites in tea, with amino acids and flavonoids showing the most significant increases.

**Figure 10:**
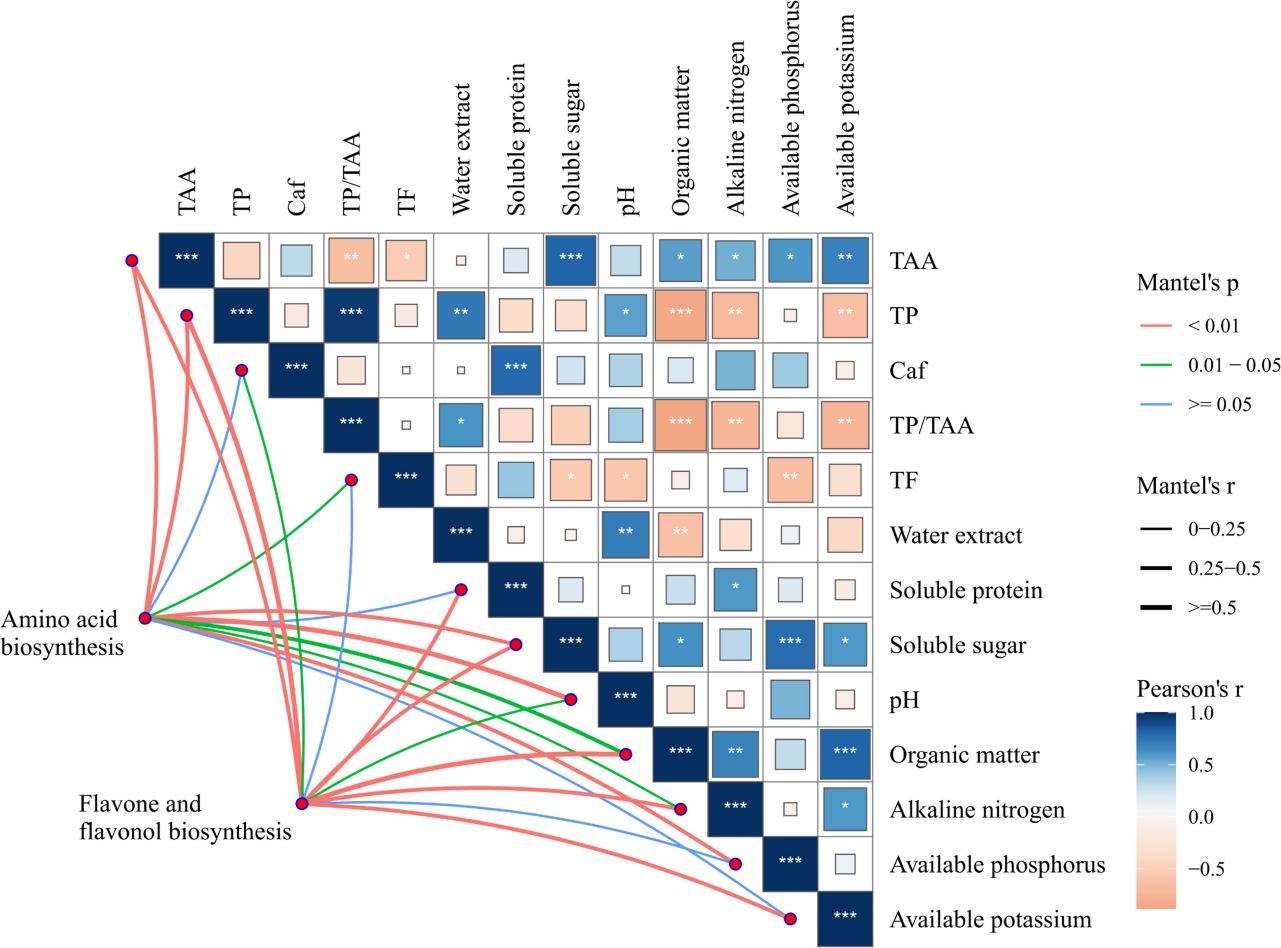
Correlation of soil nutrient and tea quality indexes. *p ≤0.05, **p ≤ 0.01, ***p ≤ 0.001, the thickness of the lines represents the magnitude of the r-value of the correlation coefficient, the depth of the color represents the correlation between the indicators.

**Figure 10** shows the correlation between soil nutrients and tea quality indices. Soil AN, AP, and AK contents showed a significant positive correlation with tea free amino acid content (*p* < 0.05), and a significant negative correlation with tea polyphenols and phenol-ammonia ratio (*p* < 0.05). These results indicated that BF application can enhance tea free amino acid levels and reduce the phenol-ammonia ratio by increasing the availability of N, P, and K in tea plantation, thereby improving the overall tea quality. Additionally, SOM, AP and AK contents were positively correlated with tea soluble sugar content, but negatively correlated with water extract content. According to the color and thickness of the lines, it is evident that free amino acids, tea polyphenols, soluble sugar, pH, organic matter, and AN are key factors significantly influencing amino acid biosynthesis, as well as flavone and flavonol biosynthesis pathways in tea leaves.

## 5 Conclusions

This study explored the effects of BF application on soil nutrient status and tea quality. After 2 years of optimized biochar-based fertilizer (BF2) treatment, significant improvements were observed in soil pH, SOM, AN, AP, and AK content. In addition, ordinary biochar-based fertilizer (BF1) application for 1 year resulted in the greatest increase in bud length, while 2 years of BF1 application led to the highest bud weight. Meanwhile, BF2 applied for 2 years had a positive impact on tea quality. Furthermore, BF2 applied for 2 years markedly enhanced the contents of free amino acids, total flavonoids and soluble sugar, and notably reducing the phenol-ammonia ratio. Untargeted metabolomic analysis revealed that BF primarily influenced amino acids and catechins, with differential metabolites mainly enriched in amino acid biosynthesis, flavonoid and flavonol biosynthesis, caffeine metabolism pathways. Moreover, prolonged BF application exerted greater benefits on tea quality improvement. These findings provide valuable insights for the rational application of BF to promote soil health and sustainable tea plantation development. Nonetheless, the influence of soil type, climatic conditions, and tea variety on fertilizer application should be carefully considered in future studies.

## Data Availability

The raw data supporting the conclusions of this article will be made available by the authors, without undue reservation.
