# Supplementary material for: Biochar-based fertilizer increases soil nutrients and enhances tea quality: a metabolomics-based analysis
Source: Front Plant Sci. 2025 May 30;16:1552759. doi: 10.3389/fpls.2025.1552759 (PMC12162341; doi:10.3389/fpls.2025.1552759)
Supplement: Supplementary file 1 [file Table1.docx]

Table S1 Basic properties of biochar-based fertilizer

| name | total nitrogen（%） | total phosphorus（%） | total potassium（%） | organic carbon（g·kg^-1^） | pH_water_ | ash（%） | moisture content（%） |
| --- | --- | --- | --- | --- | --- | --- | --- |
| BF1 | 3.18 | 0.61 | 1.74 | 216.49 | 8.05 | 24.12 | 29.34 |
| BF2 | 2.53 | 0.24 | 1.53 | 190.69 | 8.38 | 34.68 | 30.56 |
